# Oxidative Stress in the Pathogenesis and Evolution of Chronic Kidney Disease: Untangling Ariadne’s Thread

**DOI:** 10.3390/ijms20153711

**Published:** 2019-07-29

**Authors:** Anila Duni, Vassilios Liakopoulos, Stefanos Roumeliotis, Dimitrios Peschos, Evangelia Dounousi

**Affiliations:** 1 Department of Nephrology, Medical School, University of Ioannina, 45110 Ioannina, Greece; 2 Division of Nephrology and Hypertension, 1st Department of Internal Medicine, AHEPA Hospital, School of Medicine, Aristotle University of Thessaloniki, 54636 Thessaloniki, Greece; 3 Laboratory of Physiology, Medical School, University of Ioannina, 45110 Ioannina, Greece

**Keywords:** oxidative stress, albuminuria, renal fibrosis, endothelial dysfunction, inflammation

## Abstract

Amplification of oxidative stress is present since the early stages of chronic kidney disease (CKD), holding a key position in the pathogenesis of renal failure. Induction of renal pro-oxidant enzymes with excess generation of reactive oxygen species (ROS) and accumulation of dityrosine-containing protein products produced during oxidative stress (advanced oxidation protein products—AOPPs) have been directly linked to podocyte damage, proteinuria, and the development of focal segmental glomerulosclerosis (FSGS) as well as tubulointerstitial fibrosis. Vascular oxidative stress is considered to play a critical role in CKD progression, and ROS are potential mediators of the impaired myogenic responses of afferent renal arterioles in CKD and impaired renal autoregulation. Both oxidative stress and inflammation are CKD hallmarks. Oxidative stress promotes inflammation via formation of proinflammatory oxidized lipids or AOPPs, whereas activation of nuclear factor κB transcription factor in the pro-oxidant milieu promotes the expression of proinflammatory cytokines and recruitment of proinflammatory cells. Accumulating evidence implicates oxidative stress in various clinical models of CKD, including diabetic nephropathy, IgA nephropathy, polycystic kidney disease as well as the cardiorenal syndrome. The scope of this review is to tackle the issue of oxidative stress in CKD in a holistic manner so as to provide a future framework for potential interventions.

## 1. Introduction

The global burden of chronic kidney disease (CKD) is high and increases regardless of our improved understanding of the disease-associated processes on molecular, cellular, and clinical levels [1]. Aging of the population and the growing prevalence of diabetes mellitus appear to be the main culprits responsible for the CKD epidemic, and appropriate treatment of the causes and risk factors associated with disease progression is imperative yet currently not enough [1]. Several, large studies have shown that patients with CKD carry a markedly increased morbidity and mortality risk, and even mild-to-moderate elevations in serum creatinine levels are associated with high cardiovascular and overall mortality, including ischemic heart disease and heart failure syndromes [1,2].

Accordingly, cardiovascular mortality rates appear to double in patients with stage 3 CKD (estimated glomerular filtration rate (eGFR) 30–59 mL/min per 1.73 m^2^) in comparison to individuals with normal renal function, and they even triple in stage 4 (15–29 mL/min per 1.73 m^2^) [3,4,5].

Abundant experimental and clinical evidence that has accumulated during the last 3 decades has demonstrated that amplification of oxidative stress in CKD holds a key position as a central link of the intricate and intertwining pathways involved in the pathogenesis of CKD [6,7]. Excessive production of reactive oxygen species (ROS) in the setting of the activation of several enzymatic systems such as nicotinamide adenine dinucleotide phosphate (NADPH) oxidase, lipoxygenase, xanthine oxidase, uncoupled nitric oxide synthase (NOS), and the mitochondrial respiratory chain, together with impaired antioxidant defense mechanisms (i.e., superoxide dismutase (SOD), catalase, selenium-containing glutathione peroxidase, and the paraoxonases (PON)), are at the core of the imbalance between the accentuated pro-oxidant and deficient antioxidant capacity that occurs in CKD, further leading to oxidation of macromolecules, tissue damage, and dysfunction [6,7]. Thus, excess generation of ROS has been directly linked to disease mechanisms and processes associated with CKD initiation and progression, including proteinuria, arterial hypertension, and diabetes mellitus [5,6,7,8]. Additionally, the triad of oxidative stress, chronic microinflammation (which implies a state of persistent, low-grade, and subclinial augmentation of the inflammatory responses), and endothelial dysfunction as a CKD hallmark, maintain and perpetuate the vicious circle where chronic kidney damage begets more kidney injury and the systemic complications of CKD with cardiovascular dysfunction in particular [9,10,11,12,13].

Amplification of oxidative stress in CKD has been traditionally attributed to the loss of renal function and the modality of renal replacement therapy (hemodialysis or peritoneal dialysis) in patients with end-stage CKD. Thus, both higher ROS production and decreased clearance of pro-oxidant substances in the setting of renal dysfunction together with impairment of the antioxidant armamentarium are responsible for the pro-oxidant milieu that characterizes CKD [14,15,16,17,18,19].

However, it should be noted that oxidative stress is already present even in the early stages of CKD with increased NADPH oxidase-dependent superoxide production by inflammatory cells in the circulation [20,21]. Furthermore, research models of ischemia reperfusion injury suggest that oxidative stress might also serve as a link between acute kidney injury (AKI) and progression to CKD [22,23]. Biomarkers that characterize transition of AKI to CKD, such as urinary thioredoxin—a redox regulating protein—or urinary serpinA3/alpha-1-antichymotrypsin and angiotensinogen, are the subject of extensive, ongoing research [24,25].

Oxidative stress markers such as plasma F2-isoprostanes, 8-oxo-7,8-dihydro-2′-deoxyguanosine, malonyldialdehyde (MAD), advanced oxidation protein products (AOPPs) and carbamylated proteins, as well as asymmetric dimethylarginine (ADMA) and oxidized lipoprotein particles have been shown to accumulate in CKD as renal dysfunction progresses [8,13,14,26].

The scope of this review is to address the role of oxidative stress in the pathogenesis and evolution of CKD in a holistic manner and provide a future framework for potential interventions.

## 2. Implications of Oxidative Stress in Glomerular Injury and Albuminuria

Albuminuria is a well-established marker of kidney damage. It occurs early in many forms of CKD and is a major contributor to disease progression via induction of both mesangial and tubular toxicity as well as activation of intrinsic renal and systemic inflammatory pathways [27,28,29]. A dysfunctional glomerular filtration barrier with podocyte injury are at the core of proteinuria development and subsequent glomerulosclerosis [30]. Mature podocytes do not proliferate in vivo due to the highly differentiated phenotype that they possess; thus, they respond to different patterns of injury through detachment from the glomerular basement membrane, dedifferentiation, autophagy, and apoptosis [30]. The mechanisms underlying podocyte injury are complex and include hemodynamic and metabolic pathways as well as the interplay of vasoactive molecules, growth factors, and cytokines [30,31]. Accumulating experimental and clinical evidence suggests that podocytes are quite vulnerable to oxidative damage, and amplification of oxidative stress seems to be a final and common pathway shared by different aggressors at the cellular level.

Accordingly, in early experimental models of minimal change disease progressing to focal segmental glomerulosclerosis (FSGS) based on puromycin aminonucleoside, a podocyte toxin, showed that glomerular injury was directly mediated by increased generation of ROS, such as H_2_O_2_, OH¯, superoxide anion radicals, and lipid peroxidation products, by the podocytes themselves [32,33,34,35]. ROS subsequently interacted with and modified intracellular molecules with detrimental consequences, including ultrastructural changes in podocytes, defective anchoration via alteration of adhesion molecule a3b1-integrin, and DNA damage [36,37,38].

The major glomerular generators of ROS are the p47phox-containing NAPDH oxidases NOX1, NOX2, and NOX4, with the latter being selectively expressed in the kidney [39,40,41]. Both NOX1 and NOX2 require the regulatory subunit p47phox in order to exert their function and catalyze electron transfer from NADPH onto molecular oxygen, with superoxide as the end-product [39,40,41,42]. Deletion of p47phox in an experimental model of integrin-a1 double-knockout mice was associated with reduced basal levels of superoxide and collagen IV production and resulted in abolished podocyte injury, macrophage infiltration, and ensuing albuminuria and fibrosis [43].

Markers of oxidative DNA and RNA damage have been related to albuminuria even among apparently normal individuals. Accordingly, urinary levels of 8-oxo-7,8-dihydroguanosine (8-oxoGuo) were shown to be independently associated with incident low-grade albuminuria during a median follow-up of 5.6 years in 1591 participants in the Renal Iohexol Clearance Survey of the Sixth Tromsø Study not reported to suffer from renal disease, diabetes, or cardiovascular disease [44].

The key event in the development and progression of FSGS is TGF-β activation in the podocytes [45,46]. Recent data suggest that TGF-β activation is associated with accentuated crosstalk between the podocytes and the glomerular endothelium via endothelin signaling [47]. In this context, TGF-β signaling promotes synthesis of precursor molecules of endothelin in podocytes and expression of endothelin receptors by the glomerular endothelium in cell cultures [47]. The result of endothelin interaction with its receptors is suppressed mitochondrial function and induction of oxidative stress in the glomerular endothelium, with mitochondrial oxidative DNA damage becoming evident before podocyte injury [47]. Moreover, specific antagonists of endothelin-1 and its receptor or mitochondria-targeted antioxidants appear to eliminate mitochondrial oxidative stress, dysfunction of endothelial cells, and podocyte depletion in this experimental model of FSGS [47]. The mechanisms through which endothelial mitochondrial damage and cellular dysfunction promote podocyte apoptosis and progression of FSGS to overt renal failure remain to be elucidated; however, decreased levels of NO might be implicated, as occurs in the setting of diabetic nephropathy [47,48].

AOPPs are dityrosine-containing byproducts of plasma proteins, produced during oxidative stress [49,50]. AOPPs are carried in the plasma mainly by fibrinogen and albumin. They are considered as markers of oxidative stress, while a proinflammatory role of these compounds has been suggested as well [49,50,51]. Early studies showed that plasma levels of AOPPs were at the highest levels in patients with end-stage CKD undergoing renal replacement therapy (RRT). Similarly, pre-dialysis CKD patients and diabetic patients display significantly higher plasma levels compared to healthy controls [50,51].

Chronic plasma accumulation of AOPPs has been associated with podocyte loss, proteinuria, and glomerulosclerosis. Induction of the p53–Bcl-2-associated X (Bax)–caspase-3 proapoptotic pathway within the podocytes mediated by NADPH oxidase-dependent superoxide generation has been suggested as the potential pathogenic mechanism [52]. Thus, experimental data demonstrated that chronic administration of AOPPs in normal rats increased both urinary albumin and 8-hydroxydeoxyguanosine excretion, a biomarker of oxidative stress in vivo [52]. Exposure of podocytes to AOPPs induced protein kinase C (PKC)-mediated activation of total NADPH oxidase. Accordingly, expression of the key subunits of NADPH oxidase, including that of p47phox, p22phox, NOX 2, and NOX 4, was rapidly upregulated in cultured podocytes within hours of exposure to AOPPs. On the other hand, apoptosis induced by AOPPs could be blocked by pretreatment of podocytes with NADPH oxidase inhibitors and free-radical scavengers in vitro [52].

The plasma membrane receptor in podocytes, through which AOPPs induce their action, is the receptor of advanced glycation end product (RAGE), a member of the IG superfamily and a multiligand signal–transduction receptor, which has been implicated in diabetic nephropathy as well as inflammation and ischemia/reperfusion injury [53,54,55,56,57].

Furthermore, recent evidence has indicated that AOPPs promote overexpression of glucose-regulated protein 78 and CCAAT/enhancer-binding protein-homologous protein in podocytes, which is associated with production of ROS together with endoplasmic reticulum stress and, as a result, podocyte apoptosis [58].

Wnts are a family of secretory proteins that, upon binding to their cell membrane receptor, induce a series of downstream signaling events resulting in phosphorylation of β-catenin [59]. Following stabilization and activation, β-catenin translocates to the nuclei and promotes the transcription of Wnt target genes [59]. Wnt/β-catenin signaling is activated in glomerular podocytes in a wide variety of proteinuric kidney diseases and is considered to be a significant mediator of podocyte dysfunction and proteinuria [59]. Several target genes of Wnt/β-catenin that are involved in pathways of podocyte dysfunction have been identified so far. Thus, activation of Wnt/β-catenin in glomerular podocytes induces the expression of Snail1, a transcription repressor that inhibits the expression of nephrin, which is a structural component of the podocyte slit diaphragm [59,60]. Additionally, Snail1 is considered to play a key role in the epithelial–mesenchymal transition (EMT) [59,60]. As a result, these effects trigger podocyte dedifferentiation and EMT, leading to disruption of the integrity of the glomerular filtration barrier. Wnt/β-catenin suppresses Wilms tumor protein, a key transcription factor that protects the differentiated state of podocytes, and its loss is considered a hallmark of proteinuric kidney diseases [61]. Wnt/β-catenin signaling might also cause podocyte dysfunction by inducing expression of the canonical transient receptor potential cation channel 6 (TRPC6), a calcium channel that is expressed in podocytes and associated with familial and acquired forms of nephrotic syndrome [59,62]. Expression of MMP-7, an endopeptidase that degrades extracellular matrix substrates, is transcriptionally regulated by Wnt/β-catenin [59,63]. In glomerular podocytes, MMP-7 has been found to proteolytically degrade nephrin, and its expression is induced in the injured kidney [59]. Finally, in cultured podocytes, it has been shown that Wnt/β-catenin and the renin–angiotensin system (RAS) can mutually stimulate each other, maintaining a vicious cycle that leads to progressive proteinuria [59,64]. It should be noted that angiotensin II is a major stimulant for the generation of ROS [59,65].

Accumulating evidence suggests that the action of Wnt/β-catenin is dependent on the activation of RAGE by AOPPs. This triggers a cascade of reactions, including induction of NADPH oxidase, generation of ROS, and nuclear factor-κB activation, which finalizes into induction of Wnt ligands, Wnt1 and Wnt7a, and activation of β-catenin [66]. More specifically, Nox2 and p47phox, the major subunits of the NADPH oxidase complex, were upregulated in cultured podocytes after treatment with AOPPs, whereas pretreated podocytes with antioxidant compounds such as *N*-acetyl cysteine D abolished AOPP-mediated Wnt/β-catenin activation [66]. Blockage of Wnt signaling by Klotho or genetic ablation of β-catenin also protected podocytes from AOPPs [66]. In conclusion, considering that Wnt/β-catenin controls key pathways implicated in podocytopathies, targeting Wnt/β-catenin signaling might prove to be an effective approach for the development of treatment strategies in proteinuric CKD.

On the other hand, oxidative stress pathways appear to be implicated not only in the process of the initial podocyte damage and development of proteinuria, but during the chronic phase of kidney injury as well, where persisting proteinuria begets more kidney injury and progressive renal failure. Examination of the tubulointerstitial changes that occur in nephrectomized kidneys from children with congenital nephrotic syndrome of the Finnish type (NPHS1) during infancy has shown strong interstitial expression of myeloperoxidase (MPO) [67]. This enzyme is mainly produced by interstitial mononuclear cells and generates hypoclorous acid (HOCl), a potent oxidant causing irreversible tissue damage [67]. On the other hand, very low levels of free glutathione were observed in the cortex of the NPHS1 kidneys [67]. These findings together further support the fact that proteinuric kidneys are heavily submitted to oxidative stress. Additionally, severe proteinuria has been associated with increased glomerular filtration of plasminogen, which is activated to plasmin by urokinase-type plasminogen activator (uPA). Human podocytes express receptors for plasminogen and uPA. Treatment of podocytes with plasminogen has been shown to upregulate NADPH oxidase isoforms NOX2 and NOX4, and it increases production of free radicals, specifically the superoxide anion, by the mitochondria [9]. The superoxide anion promotes endothelin-1 synthesis and the expression of the β scavenger receptor CD36, resulting in podocyte apoptosis [9].

Moreover, urinary albumin undergoes endocytosis in the proximal tubule via the scavenger receptors megalin, cubilin, and CD36. Internalization of receptor–albumin complexes activates PKC and subsequently NADH oxidase-mediated ROS generation [68,69,70].

There is little evidence regarding the role of the antioxidant enzymatic system in proteinuric CKD [71,72,73]. A recent study showed that the presence of extracellular superoxide dismutase (EC-SOD) had beneficial effects in a murine model of Adriamycin-induced glomerular injury, characterized by albuminuria and renal dysfunction. EC-SOD decreased oxidative stress and inhibited NADPH oxidase upregulation and pathologic β-catenin signaling [73]. On the other hand, EC-SOD deficiency was associated with proteinuria in the setting of chronic angiotensin II infusion or increased amounts of daily albumin administration [73].

## 3. Oxidative Stress, Interstitial Fibrosis, and Chronic Kidney Disease (CKD) Progression

Renal fibrosis is the final common pathological denominator in the setting of chronic kidney injury, which occurs regardless of the primary underlying insult [74]. The extent of tubulointerstitial fibrosis is the best predictor for kidney survival in patients with CKD [74]. Renal fibrosis is characterized by excessive deposition of the extracellular matrix, which disrupts and replaces the normal kidney parenchyma and, as a result, leads to progressive loss of kidney function. The process of renal scarring, better understood in recent years, involves a complex interaction of molecular pathways, growth factors, cytokines, and cells [75,76,77,78].

Kidney myofibroblasts originating from resident renal fibroblasts and hematopoietic cells migrating into the kidney are the key collagen-producing cells involved in fibrosis [79]. TGF-β1 is the key molecule responsible for myofibroblast differentiation to a profibrotic phenotype, characterized by expression of α-smooth muscle actin (αSMA) and contractile properties [80,81]). Accordingly, TGF-β1 binds to TGF-β receptor type II on the cell surface, and subsequent TGF-β receptor type I phosphorylation of Smad2/3, which complexes with Smad4, leads to the formation of a heterodimer that translocates and binds to the promoter region of the *α-SMA* gene in the nucleus [80,81]. There is now firm evidence that TGFβ1 upregulates Nox2 and Nox4, which are both expressed by kidney fibroblasts [80,81]. Thus, both isoforms of NADPH oxidase and their byproducts such as superoxide appear to have a predominant role in the phenotypic transition of fibroblasts to myofibroblasts and fibrogenesis [80,81]). Upon TGFβ activation, it appears that Nox4 expression levels greatly exceed those of Nox2, whereas inhibition of Nox4 has been shown to substantially inhibit α-SMA and extracellular matrix production [80,81].

Nox4 has been recognized as a significant mediator in uremic toxin induced injury of the proximal kidney tubule epithelium. More specifically, experimental models indicate that p-cresyl sulfate, a uremic toxin that accumulates with CKD progression, enhances the activity of Nox4-, p22phox-NADPH, and ROS production in renal tubular cells, which in turn induces the expression of inflammatory cytokines and profibrotic factors, leading finally to reduced cell viability [80]. Likewise, indoxyl sulfate, another uremic toxin that accumulates as early as stage 3–4 CKD, has been linked not only to progression of glomerulosclerosis and interstitial renal fibrosis but to cardiac fibrosis as well [82,83].

However, it should be noted that the role of NOX4 in the generation of kidney fibrosis is not straightforward. In an experimental model of unilateral ureteral obstruction (a well-described model of renal tubular stress leading to kidney fibrosis) of wild-type and NOX4 knockout mice, deletion of NOX4 was associated with TGF-β1-mediated tubular cell apoptosis, defective hypoxia-inducible factor-1a (HIF-1a) oxygen sensing and NRF2 antioxidant pathways as well as accentuation of kidney fibrosis in obstructed kidneys [84]. In this case the antioxidant beneficial role of NOX4 might be indirect through regulation of the NRF2 pathway in kidney tubular cells. The Nrf2/Keap1 system regulates the transcription of antioxidant genes, including catalase and superoxide dismutase, through direct Nrf2 binding to responsive elements in the promoter region of target genes or via Keap1-induced NF-κB inhibition [85]. Nrf2 activity is associated with antioxidant and renoprotective effects in CKD animal models [86,87]. NRF2 under normal conditions is degraded and inactivated in proteasomes by the oxidative-stress sensor molecule Kelch-like ECH-associated protein 1 (KEAP1) [85,88]. ROS alter the conformation of KEAP1 and inactivate the interaction between KEAP1 and NRF2 [85,88]. NRF2 avoids degradation in cells exposed to oxidative stress, and after translocating into the nucleus, it activates the transcription of its target genes by binding to NRF2 recognition sequences [85,88]. It has been suggested that NOX4-related hydrogen peroxide production is an important regulator of NRF2 stability via oxidation of KEAP1 in kidney tubular cells [84,85,86,87,88].

Autophagy is the physiological process that involves degradation and recycling of intracellular components and serves as a housekeeping mechanism to remove damaged or aged macromolecules or organelles [89]. Autophagy is considered to play a protective role in both acute and chronic kidney disease [89,90]. Emerging data suggest that oxidative stress and autophagy are interconnected, and ROS together with reactive nitrogen species (RNS) induce autophagy and vice versa [89,90].

## 4. Oxidative Stress, Microvascular Dysfunction, and Chronic Inflammation in CKD

The endothelium is an essential component in the regulation and maintenance of normal renal function [91]. It is well known that the vascular endothelium is especially vulnerable to oxidative stress, and vascular oxidative stress is considered to play a critical role in CKD progression [91,92,93]. One of the most important functions of the endothelium is to secrete nitric oxide (NO), a relatively unstable diatomic free radical, involved in several biological processes including vasodilatation mediated by cyclic guanosine monophosphate (cGMP) in smooth muscle cells, inflammation, and immune responses [92]. NO is synthesized from arginine by the enzyme nitric oxide synthase (NOS), which is expressed in various isoforms—inducible (iNOS), constitutive (cNOS), neuronal (nNOS), and endothelial (eNOS)—in the endothelial cells and several other cell types [94]. Accordingly, in the renal tissue, cNOS is expressed in the vessels, glomeruli, and tubules; the iNOS isoform is mainly found in vascular smooth muscle cells (VSMCs) and the mesangium; whereas eNOS is specifically associated with the vascular endothelium [92,93,95]. In endothelial cells, eNOS is present on the cell membrane and on the cytosolic surface of the outer mitochondrial membrane [95]. NO is considered as a key molecule directly involved in the pathogenesis of oxidative stress mediated renal disease [92,93]. The relationship between NO and ROS is bidirectional, with low levels of NO in the endothelium inducing the expression of antioxidative genes and protecting renal endothelial and mesangial cells from apoptosis and fibrosis, whereas on the other hand, increased level of ROS reduce the production of endothelium-derived NO via inhibition and/or uncoupling of NOS enzymes [92,93,95]. Under normal conditions, NO within cells is suggested to inhibit cytochrome C oxidase, a mitochondrial membrane-bound terminal enzyme in the electron transfer chain, thus potentially altering mitochondrial ROS generation. The synthesis of NO in the kidney can be blocked by inhibition of the NOS with guanidine-substituted analogues of l-arginine, such as asymmetric dimethylarginine (ADMA), which accumulates in the plasma of CKD patients since the early phases of renal disease, even before glomerular filtration is significantly reduced [93,94]. The inhibition of NO synthesis in the kidney causes decreased NO bioavailability and increased production of ROS, whereas reaction of NO with superoxide generates peroxynitrites [94,96,97]. Subsequently, renal endothelial dysfunction and increased vascular resistance ensue, with loss of the ability of NO to induce vasodilation and counterbalance vasoconstrictors such Angiotensin II, endothelin-1, and sympathetic nervous system outflow. Additionally, peroxynitrites cause further tissue damage by interacting with various target molecules, including thiols, lipids, and proteins containing aromatic amino acids [94,96,97,98].

Serum ADMA and oxidative stress markers, including plasma, erythrocyte superoxide dismutase, and glutathione peroxidase, were studied in a population of patients with CKD stages 1–5, and their levels were shown to be directly associated with the stage of CKD. Glomerular filtration rate (GFR) correlated negatively with malondialdehyde and ADMA levels and positively with erythrocyte superoxide dismutase and glutathione peroxidase. Additionally, ADMA, superoxide dismutase, and oxidized low-density lipoprotein (LDL) levels were independently related to brachial artery endothelium dependent vasodilatation, implying that levels of oxidative stress and ADMA independently affect endothelial function [93].

ROS are directly involved in the signaling process that controls sodium reabsorption in the renal tubular cells and are associated with increased renal vascular resistance. Accordingly, NADPH oxidase-induced superoxide production in vascular smooth muscle cells (VSMCs) has been shown to cause vasoconstriction and increased vascular tone in the medullary circulation of mice [99,100].

Impairment of renal autoregulation, which is determined by the tubuloglomerular feedback response and myogenic contraction of the VSMC of the afferent arteriole in response to an increase in perfusion pressure, has been related to CKD progression, as impaired autoregulation augments the pressure in the glomerular capillaries and causes renal parenchymal damage with accelerated loss of kidney function. ROS, as shown by experimental studies, might mediate the impaired myogenic responses of afferent arterioles from the kidneys in CKD models [101,102,103,104,105]. Both Nox2 and Nox4 are expressed in renal resistance arteries, and accumulating data in the literature has shed light on the role of Nox2-derived ROS in the regulation of afferent arteriolar tone and renal hemodynamics [101,102]. An increase in perfusion pressure of individual afferent arterioles isolated from normal mouse kidneys increases vascular superoxide from NADPH oxidase, resulting in myogenic contraction of the afferent arteriole [101,102]. On the other hand, myogenic contractions of afferent arterioles have been found to be significantly impaired in 5/6 nephrectomized mice by hydrogen peroxide (H2O2), generated through upregulated arteriolar expression of POLDIP2/NOX4, thus counteracting the effects of superoxide and causing impaired renal autoregulation [102,103,104]. Specifically, increases in perfusion pressure of arterioles of CKD mice doubled superoxide generation while simultaneously expressing over 40% more mRNA and protein for NOX4 and POLDIP2, thus leading to a sevenfold increase in H_2_O_2_ production [103,104,105].

Oxidative stress and inflammation, as well as their interaction, are considered as the main pillars in the pathogenesis and progression of CKD [106,107,108]. Oxidative stress promotes inflammation via formation of proinflammatory oxidized lipids, AOPPs and AGEs, (advanced glycation end-products) whereas activation of nuclear factor κB (NFκB) transcription factor in the pro-oxidant milieu promotes the expression of proinflammatory cytokines as well as recruitment and activation of leukocytes and other resident proinflammatory cells [106,107,108,109,110]. Likewise, proinflammatory cytokines, such as tumor necrosis factor-α (TNFα), bind to their receptors on tubular and other renal cells and trigger signaling pathways that activate nuclear factor κB (NFκB) transcription factors [109,110]. Additionally, in the setting of chronic inflammation, activated leukocytes generate ROS, chlorine, and nitrogen species, thus accentuating and perpetuating oxidative stress [106,107,108]. Indeed, the initial ROS-mediated kidney injury triggers the ensuing renal and systemic inflammatory response.

Accordingly, in a cohort of 176 patients with CKD stage 1 to 5, serum levels of hs-CRP, interleukin-6, and malondialdehyde were significantly increased and inversely related to the GFR, whereas serum levels of superoxide dismutase and glutathione peroxidase were significantly decreased. It should be noted that IL-6 and hs-CRP were positively correlated with malondialdehyde and negatively associated with superoxide dismutase and glutathione peroxidase, further supporting the relationship between inflammation and oxidative stress in CKD [108]. It has been also shown that at high uremic concentrations, TNFα induces leukocyte oxidative bursts and increases the percentage of ROS-producing monocytes and granulocytes in the whole blood of healthy controls [111].

As already noted above, Nrf2 has a critical role for the coordinated induction of several genes encoding antioxidant enzymes, thus maintaining a redox balance in the organism. Additionally, several studies have demonstrated the anti-inflammatory properties of Nrf2 through suppression of inflammatory genes such as those encoding TNF-alpha-induced monocyte chemoattractant proteins (MCP)-1 and VCAM-1. Dysfunctional Nrf2 activation as occurs in CKD renders the kidney vulnerable to the effects of oxidative stress and, at the same time, potentiates intra-renal inflammation by promoting accumulation of hydroperoxides and lipoperoxides, which are potent activators of NF-κB [112,113,114]. Studies conducted in animals with 5/6 nephrectomy-induced CKD have shown a prominent reduction in nuclear Nrf2 content together with marked elevation of the repressor molecule Keap1, suggesting an impaired regulation of this antioxidant mechanism despite the presence of oxidative stress and inflammation, which should have induced Nrf2 activation [112,113,114,115].

## 5. Oxidative Stress and Clinical Models of CKD

Diabetic kidney disease continues to be on the rise and represents the leading cause of end-stage kidney disease [116]. It is the interplay between metabolic and hemodynamic factors that sets in motion a set of intertwining signals and pathways, involving, among others, amplification of oxidative stress and inflammation [116,117,118]. An elaborate description of the intricate mechanisms operating in the pro-oxidant environment created by hyperglycemia is beyond the scope of this review. However, it should be noted that the clinical and experimental evidence regarding amplification of oxidative stress in diabetic nephropathy is immense and has provided the main matrix upon which we base our current knowledge about the molecular mechanisms involved in oxidative stress induction and its adverse consequences in patients with kidney disease.

Hyperglycemia, AGEs together with activation of RAAS, and the ensuing glomerular hyperfiltration promote increased generation of ROS by upregulation of Nox4 as well as TGF-β1 expression in the glomerular cells of diabetic kidneys [118].

ROS, in return, induce signaling pathways, including the nuclear factor-kappaB (NF-κB) pathway, p38 mitogen-activated protein kinase (MAPK), Jun N-terminal kinases/stress activated protein kinases, as well as eNOS uncoupling together with increased expression of vascular endothelial growth factor (VEGF) and of inflammatory cytokines [42,119,120,121,122,123,124]. In diabetic rats, augmented oxidative stress has been demonstrated to significantly increase monocyte chemotactic protein-1 (MCP-1) levels and stimulate macrophage recruitment, mainly through activation of the PKC pathway [125].

Recent data indicate that mitochondria of diabetic patients display increased fragmentation, the so-called mitochondrial fission, which has been associated with mitochondrial dysfunction, increases in ROS production, and subsequent endothelial damage [126,127,128]. Additionally, ROS cause DNA modifications leading to further mitochondrial and cellular damage, triggering of apoptotic pathways through the activation of caspases as well as expression of p53 proteins, and eventually cell death [129,130]. Accordingly, early studies have indicated that urinary excretion of hydroxy-2-deoxyguanosine (8-OHdG), a marker of oxidative DNA damage, is increased in patients with diabetic nephropathy [129,130].

IgA nephropathy (IgAN), the most common glomerular disease globally, is a leading cause of CKD and renal failure. A systemic pro-oxidant environment—marked by increased serum levels of lipoperoxide or malondialdehyde and reduced activity of superoxide dismutase, catalase, and glutathione peroxidase, as detected in sera of patients with IgAN—has been associated with disease activity and progression [131,132,133]. Markedly, circulating AOPP have been shown to correlate with proteinuria and be a potent risk marker of decline in the renal function and disease progression since early in the clinical course of IgAN. AOPPs, apart from podocyte injury, can activate NF-κB and trigger transcription of iNOS, a powerful mediator of oxidative stress in mesangial cells [134].

Autosomal dominant polycystic kidney disease (ADPKD) is the most common genetic disease leading to end-stage CKD. Experimental and clinical data suggest that oxidative stress may be involved in the progression of CKD in the setting of ADPKD [135,136,137,138]. Upregulation of heme oxygenase-1 mRNA and accumulation of lipid peroxidation products, such as malondialdehyde and 4-hydroxy-2(E)-nonenal, in the plasma and kidneys together with downregulation of antioxidant enzyme mRNA expression, including glutathione peroxidase, catalase, glutathione S-transferase, and superoxide dismutase, have been observed in animal models of PKD, which also correlated with disease severity [135].

Markedly elevated markers of oxidative stress, such as plasma 8-epi-prostaglandin F2α, and attenuated antioxidant mechanisms represented by diminished SOD have been demonstrated in subjects with ADPKD since early in the disease process, before manifestation of arterial hypertension or impairment of renal function becomes evident [136].

Oxidative stress-responsive kinase-1 (OSR-1), which is associated with regulation of Na^+^ transport and cell volume during oxidative stress, has recently been shown to play a role in the regulation of blood pressure in ADPKD [139]. Thus, serum OSR-1 gene expression has been found to be significantly increased in hypertensive ADPKD patients in comparison with normotensive ADPKD counterparts, non-ADPKD hypertensive subjects, as well as healthy individuals [139].

Cardiorenal syndrome refers to the reciprocal pathophysiological interaction between heart disease and kidney disease where acute or chronic dysfunction of either organ causes acute or chronic dysfunction of the other [140]. Specifically, type 4 cardiorenal syndrome is defined as CKD causing left ventricular hypertrophy (LVH) and congestive heart failure [140].

LVH is a prevalent trait of CKD, becoming more common with CKD progression, and is significantly associated with cardiovascular events and mortality in these patients [141]. Amplification of oxidative stress has recently come into the spotlight as a potential mediator implicated in the pathogenesis of LVH and cardiorenal syndrome [13,142,143,144]. Activation of cardiac and renal NADPH oxidases, dysfunctional mitochondrial chain, xanthine oxidase, eNOS uncoupling, and nitrosative stress are the main pathways implicated in production of ROS leading to inflammation, apoptosis, and fibrosis in both the heart and the kidney [145,146,147,148,149,150]. ROS activate a broad variety of hypertrophy signaling kinases and transcription factors, such as MAP kinase (MAPK) and nuclear factor-κB (NF-κB), as well as proliferation and activation of matrix metalloproteinases (MMPs) [151]. A positive correlation has been shown between serum levels of oxidized LDL and protein carbonyl groups and left ventricular mass in children with CKD, whereas serum carbamylated albumin has been strongly associated with a four-year risk of death from congestive HF in diabetic patients with end-stage CKD [152,153]. A recent study demonstrated that polymorphisms in the silent information regulator gene 1 (Sirt1), which act as a fundamental mediator in the response to oxidative stress and inflammation, is associated with LV concentric growth in CKD patients [154]. On the other hand, dysfunctional antioxidant defenses may aggravate oxidative stress-related cardiovascular dysfunction in CKD. Thus, genetic polymorphism of the *PON1* gene have been significantly related to the severity of LVH and LV dysfunction in patients with CKD [155].

## 6. The Future of Therapeutic Strategies for Oxidative Stress Modulation

Considering the implication of oxidative stress as a causative factor in CKD progression as well as the associated spectrum of systemic complications, especially cardiovascular morbidity, it could be expected that targeted therapies would translate into overt clinical benefits. Nevertheless, except for the multipotential benefits of RAS blockade, results for the antioxidant therapies utilized until now, as shown by a Cochrane database systematic review (vitamin E, coenzyme Q, acetylcysteine, bardoxolone methyl, human recombinant superoxide dismutase), have been disappointing [156].

Recently, new medications have come into the spotlight, which apart from offering evident nephroprotection and cardioprotection are also suggested to exert antioxidant effects. Thus, sacubitril/valsartan, an angiotensin-receptor neprilysin inhibitor, as well as sodium–glucose cotransporter 2 (SGLT2) inhibitors have been shown to possess antioxidant, anti-inflammatory, and antifibrotic properties [157,158]. Future clinical trials will determine the efficacy of these or other new drugs in modulating the pro-oxidant milieu of CKD.
